# Data on cloning, expression and biochemical characteristics of a chondroitin sulfate/dermatan sulfate 4-*O*-endosulfatase

**DOI:** 10.1016/j.dib.2023.109139

**Published:** 2023-04-10

**Authors:** Lin Wei, Yingying Xu, Min Du, Ying Fan, Ruyi Zou, Xiangyu Xu, Qingdong Zhang, Yu-Zhong Zhang, Wenshuang Wang, Fuchuan Li

**Affiliations:** a National Glycoengineering Research Center, Shandong Key Laboratory of Carbohydrate Chemistry and Glycobiology and State Key Laboratory of Microbial Technology, Shandong University, 72 Binhai Rd, Qingdao 266237, China; b Qingdao Special Servicemen Recuperation Center of PLA Navy, Qingdao 266071, China; c School of Life Science and Technology, Weifang Medical University, 7166 Baotong West Street, Weifang 261053, China; d College of Marine Life Sciences, Ocean University of China, Qingdao, China

**Keywords:** Sulfatase, Cloning and expression, Biochemical characteristics, Substrate specificity

## Abstract

The data shown in this article are related to the published paper entitled “A novel 4-*O*-endosulfatase with high potential for the structure-function studies of chondroitin sulfate/dermatan sulfate” in *Carbohydrate Polymers*. In this article, the phylogenetic analysis, cloning, expression, purification, specificity and biochemical characteristics of the identified chondroitin sulfate/dermatan sulfate 4-*O*-endosulfatase (endoBI4SF) are described in detail. The recombinant endoBI4SF with a molecular mass of 59.13 kDa can can specifically hydrolyze the 4-*O*- but not 2-*O*- and 6-*O*-sulfate groups in the oligo-/polysaccharides of chondroitin sulfate/dermatan sulfate and show the maximum reaction rate in 50 mM Tris-HCl buffer (pH 7.0) at 50°C, which can be a very useful tool for the structural and functional studies of chondroitin sulfate/dermatan sulfate.

**Specifications Table**

**Table:** 

Subject	Biochemistry and Molecular Biology
Specific subject area	Phylogenetic analysis, cloning, expression, purification, specificity analysis and biochemical characteristics of the chondroitin sulfate/dermatan sulfate 4-*O*-endosulfatase endoBI4SF.
Type of data	Table Graph Figure
How the data were acquired	The protein sequence (GenBank^TM^ accession number: EDV06292.1) of endoBI4SF was downloaded from NCBI database. Other data were acquired following the “*Experimental Design, Materials, and Methods*” described in this article.
Data format	Raw Analyzed Filtered
Description of data collection	Sequence alignment and phylogenetic analysis were performed using Bio-Edit version 7.0.5.3 and MEGA version 7.0. The full-length gene of endoBI4SF without predicted signal peptide was cloned into the expression vector pET22b (+). EndoBI4SF was induced and expressed in *E. coli* BL21 (DE3), and purified with a Nickel-Sepharose^TM^ 6 Fast Flow nickel affinity column. The substrate specificity of endoBI4SF was determined using four unsaturated chondroitin sulfate disaccharides with different sulfation patterns. The optimal temperature, optimal pH, effects of metal ions/other chemicals (5 mM), thermostability and kinetic parameters were determined using 4-*O*-sulfated unsaturated CS disaccharide as substrate.
Data source location	This data was collected and analyzed at National Glycoengineering Research Center, Shandong university, Qingdao, China. Institution: National Glycoengineering Research Center, Shandong university. City/Town/Region: 72 Binhai Rd, Qingdao. Country: China.
Data accessibility	Repository name: Mendeley Data identification number: doi:10.17632/tz6yvbhzt2.1 Direct URL to data: https://data.mendeley.com/datasets/tz6yvbhzt2/1
Related research article	L. Wei, Y. Xu, M. Du, Y. Fan, R. Zou, X. Xu, Q. Zhang, Y. Zhang, W. Wang, F. Li, A novel 4-*O*-endosulfatase with high potential for the structure-function studies of chondroitin sulfate/dermatan sulfate, *Carbohyd. Polym.* 120508 (2023), 305. 10.1016/j.carbpol.2022.120508

## Value of the Data

• The data provide the detailed supplementary materials for the paper entitled “A novel 4-*O*-endosulfatase with high potential for the structure-function studies of chondroitin sulfate/dermatan sulfate” published in the journal of “*Carbohydrate Polymers*”.
• The data benefit the researchers working in the CS/DS sulfatase-related fields.
• The data provide the preparation method and biochemical parameters of the endoBI4SF for its further study and application.

## 1 Introduction

The data is supplementary of the paper entitled “A novel 4-*O*-endosulfatase with high potential for the structure-function studies of chondroitin sulfate/dermatan sulfate” in *Carbohydrate Polymers* [1], describes the specific activity, biochemical characteristics of endoBI4SF, and the methods of cloning, expression, purification and specificity analysis.

## 2 Data Description

### 2.1 Gene and Protein Sequences of endoBI4SF

**Fig. 1:**
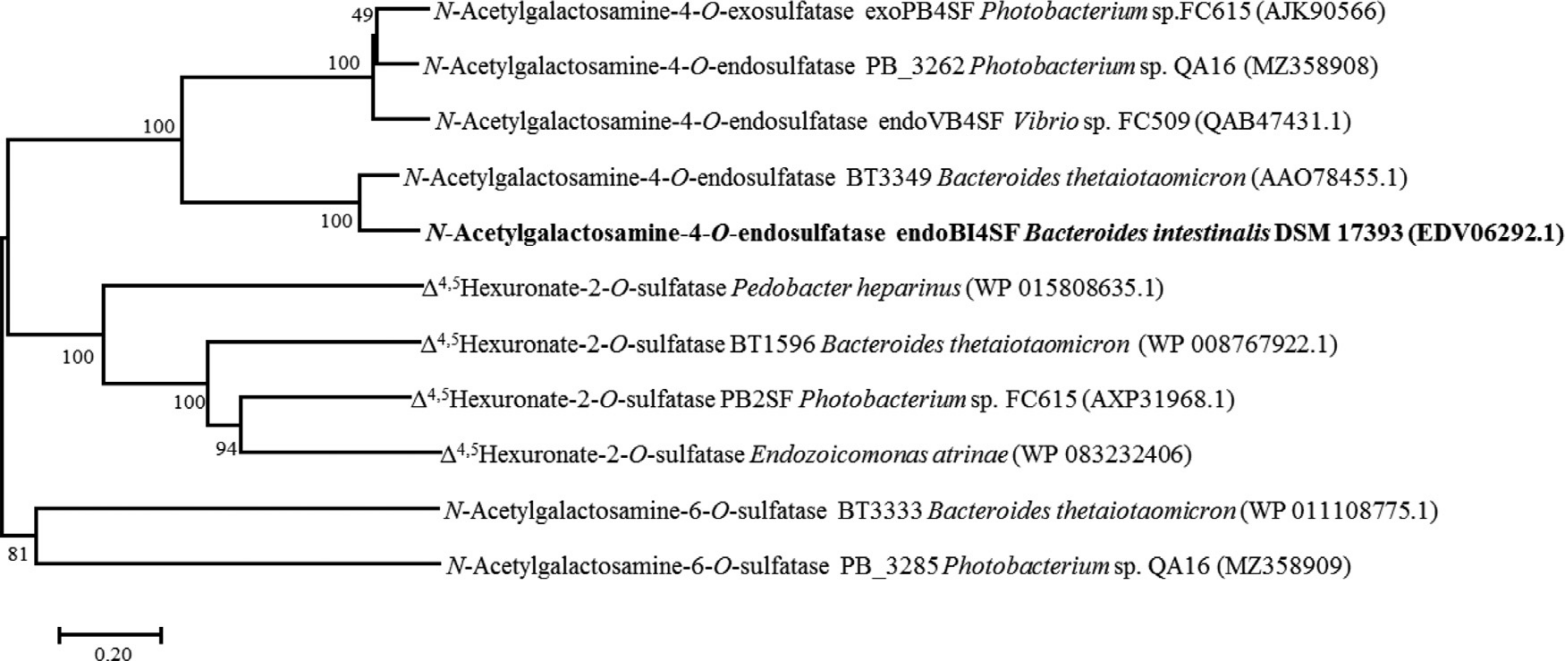
Phylogenetic analysis of endoBI4SF.

The endoBI4SF gene (GenBank accession number: EDV06292.1) is 1,548 bp in length with 45.28% GC content and encodes a protein comprising 515 amino acid residues with a 20 amino acid type II N-terminal signal peptide (Met^1^-Gly^20^). The endoBI4SF protein has a predicted molecular weight of 59.13 kDa and a theoretical isoelectric point of 5.51. According to BLASTp multiple sequence alignment, endoBI4SF is annotated as an arylsulfatase. Furthermore, phylogenetic analysis showed that endoBI4SF clustered with D-*N*-acetylgalactosamine (GalNAc) -4-*O*-sulfatases, and thus, it was preliminarily projected to be a GalNAc-4-*O*-endosulfatase (Fig. 1). All of the sequence constructed phylogenetic trees are listed in the dataset files in the repository [2].

Phylogenetic analysis of endoBI4SF was executed based on Clustal W multiple alignments with identified CS/DS sulfatases from bacteria. The phylogenetic tree was constructed by MEGA version 7.0.26 with the neighbor-joining statistical method. The percentage of replicate trees, in which the associated taxa clustered together in the bootstrap test (1,000 replicates), is shown next to the branches.

### 2.2 Heterologous Expression and Purification of endoBI4SF

**Fig. 2:**
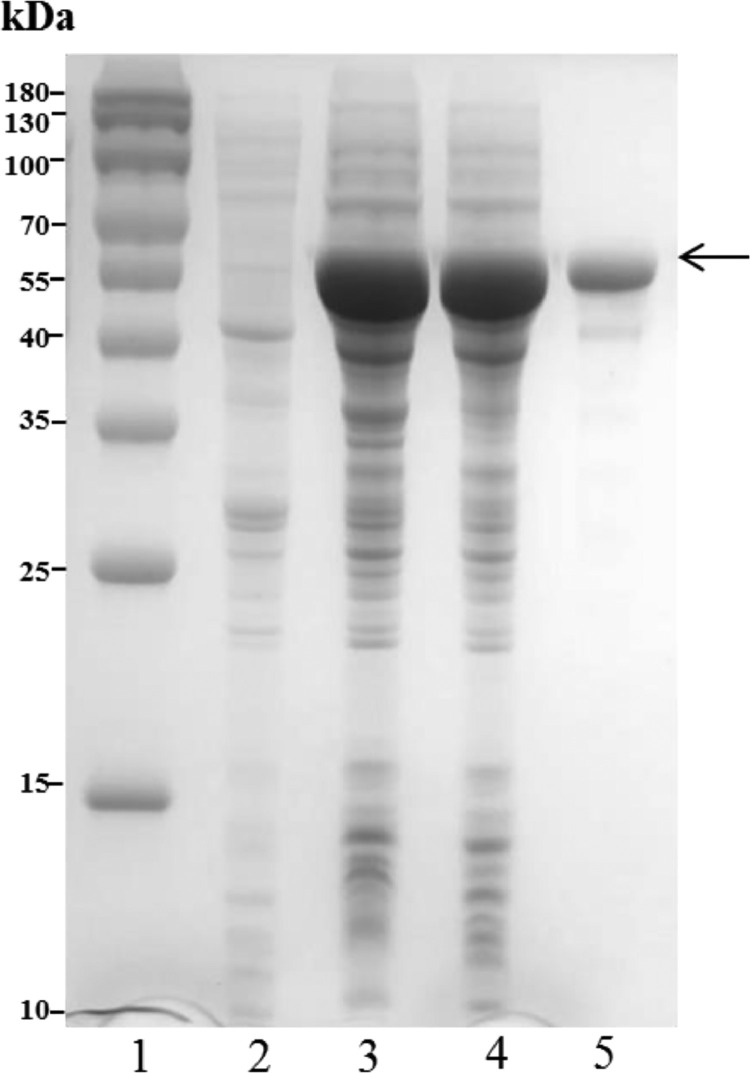
Purification of the recombinant endoBI4SF from *E. coli* by Ni^2+^ affinity chromatography.

The gene of the endoBI4SF without signal peptide sequence was amplified from the genomic DNA of *Bacteroides intestinalis* DSM 17393. The putative sulfatase fragment was cloned into a pET22b expression vector with a C-terminal His_6_ tag and then transferred into *E. coli* BL21 (DE3) cells. Cells harboring the expression vector (pET22b-endoBI4SF) were cultured and induced as described in “*Materials and methods*”. According to the SDS-PAGE, the putative endoBI4SF was successfully expressed and formed a 59 kDa soluble protein in the supernatant (Fig. 2), and the protein was approximately 90% pure after Ni^2+^ affinity chromatography purification. The expression and purification data of the recombinant protein are shown in Table 1.

**Table 1:** Purification of recombinant 4-*O*-endosulfatase

4-*O*-endosulfatase				On ΔA		On CS-A	
	Total protein (g)	protein conc. (mg/ml)	Yield (%)	Total Activity (U)	Specific Activity (U/mg)	Total Activity (U)	Specific Activity (mU/mg)
Crude protein	0.7	7	100%	-	-	-	-
Elution from Ni^2+^ column	0.12	60	17.1%	2103.6	17.53	1915.2	15.96

Expression and purification of the recombinant endoBI4SF were presented by SDS-PAGE using 13.2% polyacrylamide gels followed by staining with coomassie brilliant blue. 1, prestained protein marker (MP102) (Vazyme); 2, lysate transfected with empty pET22b plasmid; 3, lysate transfected with pET22b-endoBI4SF expression plasmid and induced with IPTG; 4, lysate supernatant transfected with pET22b-endoBI4SF expression plasmid and induced with IPTG; 5, purified recombinant endoBI4SF (2 μg) with Ni^2+^ affinity chromatography. Molecular weight markers and their corresponding masses are indicated.

### 2.3 Substrate Specificity Analysis of endoBI4SF Toward CS/DS Disaccharides

**Fig. 3:**
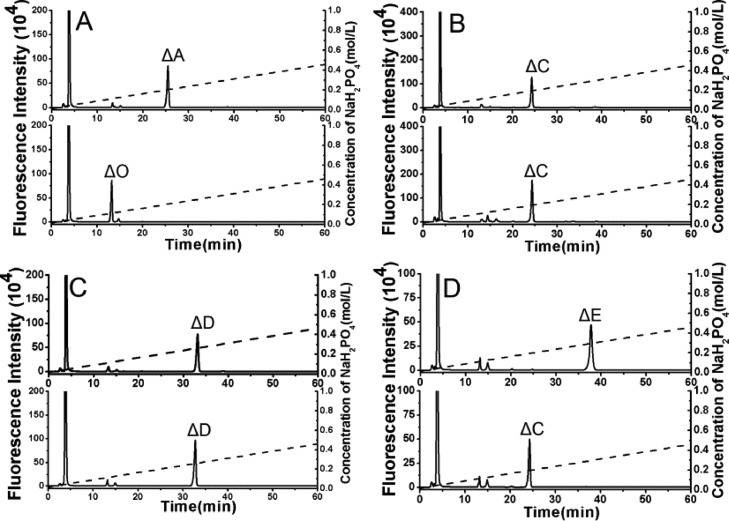
Product analysis of endoBI4SF toward various unsaturated CS/DS disaccharides.

A series of unsaturated CS/DS disaccharides with different sulfation patterns, including Δ^4,5^HexUA1–3GalNAc(4-*O*-sulfate) (ΔA), Δ^4,5^HexUA1–3GalNAc(6-*O*-sulfate) (ΔC), Δ^4,5^HexUA(2-*O*-sulfate)1–3GalNAc(6-*O*-sulfate) (ΔD) and Δ^4,5^HexUA1–3GalNAc(4, 6-O-sulfate) (ΔE), where Δ^4,5^HexUA and GalNAc represent a double bond between C4 and C5, hexuronic acid and D-*N*-acetylgalactosamine, respective, were used as substrates to preliminarily determine the specificity of endoBI4SF. EndoBI4SF was capable of hydrolyzing the 4-*O*-sulfate groups from ΔA and ΔE to produce ΔO (Δ^4,5^HexUA1–3GalNAc) and ΔC, respectively, but did not affect the 6-*O*-sulfate and 2-*O*-sulfate groups in ΔC and ΔD (Fig. 3), the putative sulfatase is classifieds as a GalNAc-4-*O*-sulfatase. The raw data used for preparing the figures has been stored in a dataset file of Mendeley Data [2].

Unsaturated CS/DS disaccharides ΔA (A), ΔC (B), ΔD (C) and ΔE (D) were digested with inactivated (*top*) or active (*bottom*) endoBI4SF. All of the products were labeled with 2-AB and then analyzed using anion-exchange HPLC. The elution positions of the standard disaccharides are indicated: ΔO (Δ^4,5^HexUAβ1–3GalNAc), ΔA (Δ^4,5^HexUAβ1–3GalNAc(4S)), ΔC (Δ^4,5^HexUAβ1–3GalNAc(6S)), ΔD (Δ^4,5^HexUA(2S)β1–3GalNAc(6S)) and ΔE (Δ^4,5^HexUAβ1–3GalNAc(4S,6S)).

### 2.4 Biochemical Characteristics of endoBI4SF

**Fig. 4:**
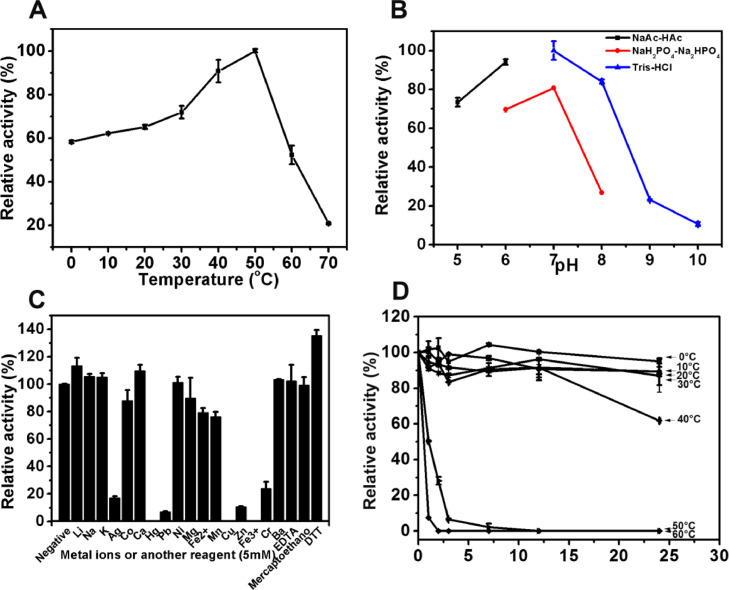
Biochemical characteristics of endoBI4SF.

The biochemical characteristics of endoBI4SF, including the optimal temperature, optimal pH, effect of metal ions or other reagents and thermostability, were estimated using ΔA as a substrate, and the raw data could be found in a dataset file of Mendeley Data [2]. As shown in Fig. 4, endoBI4SF showed the maximum reaction rate at 50°C but the activity was sharply reduced by increasing the temperature to 60°C (Fig. 4A). EndoBI4SF showed relatively high activity at pH 5.0-8.0, had its maximum reaction rate in 50 mM Tris-HCl buffer (pH 7.0) and it also retained a high activity in 50 mM NaAc-HAc buffer (pH 6.0) (Fig. 4B). Li^+^ and Ca^2+^ slightly enhanced the enzyme activity. Additionally, the univalent metal ion Ag^+^ and most divalent metal ion, such as Hg^2+^, Pb^2+^, Cu^2+^, Zn^2+^, and another metal ion like Fe^3+^ and Cr^3+^ strongly inhibited the activity of endoBI4SF. The reducing reagent DTT (5 mM) promoted the activity of endoBI4SF, but the chelating reagent EDTA showed no significant effect (Fig. 4C).

The thermostability of endoBI4SF was investigated by measuring the residual activity at the optimal conditions (50 mM Tris-HCl, pH 7.0) at 50°C after preincubating of the enzyme at a series of temperatures for 0-24 h. The enzyme activities maintain greater than 60% of its enzymatic activity even after incubation at 40°C for 24 hours and quickly decreased within 3 hours after increasing temperature to 50°C (Fig. 4D).

**Fig. 5:**
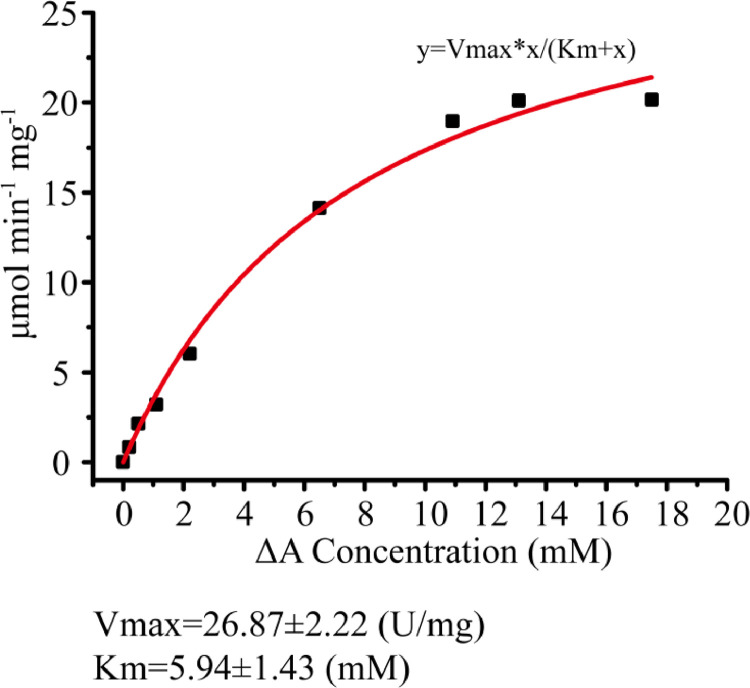
Kinetic analysis of endoBI4SF toward ΔA.

The enzymatic kinetics was also calculated using ΔA as substrate under the optimal conditions by fitting the Michaelis-Menten equation with origin version 9.6. The V_max_ and K_m_ of endoBI4SF were 26.87±2.22 (U/mg) and 5.94±1.43 (mM), respectively (Fig. 5). The raw data for preparing the figures can be found in a dataset file of Mendeley Data [2].

The optimal conditions, including effects of temperature (A), effects of pH (B), effects of metal ions, chelating reagent and reducing reagent (C) and thermostability (D) of endoBI4SF were determined using ΔA (1 mg/ml) as substrate. Error bars represent averages of duplicates ± S.D.

Reactions were performed triplicate using various concentration of ΔA with endoBI4SF (3 μg) in 50 mM Tris-HCl buffer (pH 7.0) at 50°C. The Michaelis-Menten equation was fitted using origin version 9.6.

## 3 Experimental Design, Materials and Methods

### 3.1 Materials

PrimeSTAR^TM^ HS DNA polymerases for PCR amplification were purchased from Takara Inc. (Dalian, China). The plasmid named pET22b-rVAR2 containing the gene of ID1-ID2a domain of VARCSA (rVAR2) from *Plasmodium falciparum* was synthetized by Genewiz (Suzhou, China). CS-A from bovine trachea, CS-C from shark cartilage, commercial chondroitinase ABC (CSase ABC) from *Proteus vulgaris* and 2-aminobenzamide (2-AB) were purchased from Sigma-Aldrich Inc. The competent cells *E. coli* BL21 (DE3) and Fast Mutagenesis Kit V2 were purchased from Vazyme (Nanjing, China). The sulfated CS disaccharides with various sulfation patterns, including Δ^4,5^HexUA1–3GalNAc(4S) (ΔA), Δ^4,5^HexUA1–3GalNAc(6S) (ΔC), Δ^4,5^HexUA(2S)1–3GalNAc(6S) (ΔD) and Δ^4,5^HexUA1–3GalNAc(4S,6S) (ΔE), were prepared by completely digesting several CS polysaccharides treated with CSase ABC, and separated by anion exchange High Performance Liquid Chromatography (HPLC) using a YMC-Pack PA-G column (YMC, Japan) [3].

### 3.2 Sequence Analysis of Gene and Protein of endoBI4SF

The protein sequence (GenBank^TM^ accession number: EDV06292.1) of endoBI4SF was downloaded from NCBI database. Online similarity analysis was carried out using the Protein BLAST online (https://blast.ncbi.nlm.nih.gov/Blast.cgi). The signal peptide prediction was performed by SignalP 5.0 server (https://services.healthtech.dtu.dk/service.php?SignalP-5.0). Sequence alignment and phylogenetic analysis were performed using MEGA version 7.0. The molecular mass of the putative protein was estimated using Compute pI/MW tool on the ExPASy server of the Swiss Institute of Bioinformatics (https://www.expasy.org/). Sequence alignment and phylogenetic analysis were performed using Bio-Edit version 7.0.5.3 and MEGA version 7.0.

### 3.3 Heterologous Expression and Purification of Recombinant endoBI4SF, rVAR2 and rVAR2-stGFP

The full-length gene of endoBI4SF without signal peptide sequence was amplified using high-fidelity PrimeSTAR^TM^ HS DNA polymerases (Takara, Dalian) and corresponding primer pairs with restriction enzyme sites (Table 2). PCR products were inserted into the NdeI/XhoI restriction enzyme sites of expression vector pET22b (+). Fluorescent probe rVAR2-stGFP, rVAR2 fused with stGFP on 3’-terminal, was used to specifically recognize 4-*O*-sulfated CS-A. The sequence with homologous recombination regions of stGFP was amplified and recombine with Xho I treated pET22b-rVAR2 using Fast Mutagenesis Kit V2 (Vazyme, Nanjing). The recombinant plasmid pET22b-endoBI4SF, the synthetized plasmid pET22b-rVAR2, and the fusion plasmid pET22b-rVAR2-stGFP were transformed into *E. coli* BL21 (DE3), respectively. The fragment integrities were confirmed by DNA sequencing. To express endoBI4SF, rVAR2 and rVAR2-stGFP, *E. coli* cells harboring respective expression vector were expanded in LB broth until the A_600_ values reached 0.8–1.0 at 37°C, and then induced and expressed at 16°C by supplementing with a final concentration of 0.05 mM Isopropyl 1-thio-β-D-galactopyranosid. After 24 h, the cells were collected by centrifugation at 8,000 × g, suspended with buffer A (50 mM Tris-HCl, 150 mM NaCl (pH 8.0)) and then disrupted by sonication (40 repetitions, interval 4 s stop 8 s) in an ice-cold environment. Cell lysate was separated by centrifugation at 15,000 × g for 30 min at 4°C. The supernatant containing recombination protein were loaded on a nickel affinity column with Nickel-Sepharose^TM^ 6 Fast Flow (GE Healthcare, Sweden), washed with buffer A containing 10 mM imidazole to remove impurities, and then eluted with buffer A containing 250 mM imidazole to collect the target proteins. After desalting with an Amicon Ultra 0.5-ml 10K unit (Millipore) to remove the high concentration of salt, the purified proteins were exchanged to PBS buffer and analyzed by SDS-polyacrylamide gel electrophoresis (SDS-PAGE) followed by staining with Coomassie Brilliant Blue R-250. The concentration of purified protein was determined using BCA Protein Assay Kit (Cwbio, Shanghai).

**Table 2:** Bacterial strains, plasmids, and primers.

Strains and plasmids Strains	Description	Source
*Bacteroides intestinalis* DSM 17393	Intestinal microorganisms isolated from human faces	Leibniz Institute DSMZ-German Collection of Microorganisms and Cell Cultures GmbH
*E. coli* BL21 (DE3)	F^−^*omp*T *hsd*S (rB^−^, mB^−^) *gal dcm* (DE3)	Vazyme Biotech
Plasmids		
pET22b	Expression vector; Amp^r^	Novagen
pET22b-endoBI4SF	pET22b loading an NdeI-endoBI4SF-XhoI fragment encoding the recombinant endoBI4SF fused with a His_6_ tag at the C-terminus	
pET22b-rVAR2	pET22b loading an NdeI-rVAR2-XhoI fragment encoding the rVAR2 fused with a His_6_ tag at the C-terminus	Genewiz
pET22b-rVAR2-stGFP	pET22b loading an NdeI-rVAR2-stGFP-XhoI fragment encoding the rVAR2-stGFP fused the rVAR2 with stGFP and a His_6_ tag at the C-terminus	
Primers		
endoBI4SF-F	5’-CATATGTTGCAAGGCTGCAAAACCC-3’	Sangon Biotech
endoBI4SF-R	5’-CTCGAGGTAAGGTATCATGTCGG-3’	
rVAR2-stGFP-F	5’-AGCAGCAAACTGGATCTCGAGATGGGTCACCATCATCATCA-3’	
rVAR2-stGFP-R	5’-GTGGTGGTGGTGGTGGTGGAATTCTTTGTACAGTTCATCCATGC-3’	

Restriction enzyme sites are underlined. Amp^r^, ampicillin-resistant.

### 3.4 Substrate Specificity of endoBI4SF Toward CS Disaccharides

To determine the substrate specificity of endoBI4SF, four unsaturated CS disaccharide substrates (2 nmol) with different sulfation patterns (ΔA, ΔC, ΔD and ΔE) were incubated with endoBI4SF (2 μg) in 50 mM Tris-HCl buffer (pH 7.0) at 30°C overnight. After inactivation at 85°C for 10 min, cooled in ice-cold water and centrifugation at 15,000 × g for 10 min, the supernatants of endoBI4SF-treated CS disaccharides were collected and labeled with 2-AB [4]. After extraction with chloroform to remove the free 2-AB, all the labeled samples were detected via anion exchange HPLC with a YMC-Pack PA-G column (YMC, Japan) and eluted with a linear gradient from 16 to 460 mM NaH_2_PO_4_ over 60 min at a flow rate of 1.0 ml/min at room temperature. The products were monitored with a fluorescent detector with excitation and emission wavelengths of 330 and 420 nm, respectively.

### 3.5 Biochemical Characterization of Recombinant endoBI4SF

To determine the optimal temperature of endoBI4SF, the effect of temperature toward endoBI4SF (about 0.2 μg) were tested with 30 μg ΔA (1 mg/ml) in 50 mM Tris-HCl (pH 7.0) at temperatures from 0 to 70°C for 5 min. The effects of pH were determined using a series of buffers with different pH ranges, including NaAc-HAc buffer (50 mM, pH 5.0–6.0), NaH_2_PO_4_-Na_2_HPO_4_ buffer (50 mM, pH 6.0–8.0), and 50 mM Tris-HCl buffer (pH 7.0–10.0) in a total volume of 30 μl at optimal temperature for 5 min. The effects of metal ions/other chemicals (5 mM) toward endoBI4SF were investigated by incubating with ΔA and enzyme at the optimal temperature and pH as described above. To determine the thermostability of endoBI4SF, purified enzyme was pre-incubated at temperature from 0 to 60°C for 0-24 hours, respectively, and the residual activity of enzyme was calculated by incubating the reaction mixture for 5 min in optimal condition. All reactions were carried out duplicate. The enzymatic activities were determined by gel filtration HPLC using a Superdex^TM^ Peptide 10/300 GL column with 0.20 M NH_4_HCO_3_ as mobile phase at a flow rate of 0.4 ml/min. The absorbance at 232 nm was monitored using a UV detector and analyzed online by using the software LCsolution version 1.25 [5,6]. In addition, the reaction rate of endoBI4SF against ΔA at the final concentrations of 0-25 mM was measured under the optimal conditions to analyze the enzyme kinetics. The kinetic parameters were calculated based on the Michaelis-Menten equation fitting with origin version 9.6.

## Ethics Statements

These data does not involved in human subjects, animal experiments, or data collected from social media platforms.

## CRediT Author Statement

**Lin Wei, Yingying Xu:** Methodology, Investigation, Data analysis, Writing-original draft, Draft preparation. **Min Du:** Investigation, Data curation. **Ying Fan:** Data analysis, Writing reviewing. **Ruyi Zou:** Data analysis. **Xiangyu Xu:** Investigation. **Qingdong Zhang:** Investigation. **Wenshuang Wang:** Writing reviewing. **Fuchuan Li:** Supervision, Writing reviewing, Editing.

## Declaration of Competing Interest

The authors declare that they have no known competing financial interests or personal relationships that could have appeared to influence the work reported in this paper.

## Data Availability

Original data of phylogenetic analysis, cloning, expression, purification, specificity and biochemical characteristics of the identified chondroitin sulfate/dermatan sulfate 4-O-endosulfatase (endoBI4 (Original data) (Mendeley Data).
